# Comparison of Pregnancy Outcome between Ultrasound- Guided Tubal Recanalization and Office-Based Microhysteroscopic Ostial Dilatation in Patients with Proximal Blocked Tubes

**DOI:** 10.22074/ijfs.2015.4608

**Published:** 2015-12-23

**Authors:** Emaduldin Mostafa Seyam, Momen Mohamed Hassan, Mohamed Tawfeek Mohamed Sayed Gad, Hazem Salah Mahmoud, Mostafa Gamal Ibrahim

**Affiliations:** 1 Department of Obstetrics and Gynecology, College of Medicine, El Minya University, Minya, Egypt; 2 Department of Obstetrics and Gynecology, Al Fayoum General Hospital, Faiyum, Egypt

**Keywords:** Fallopian Tube, Fertility, Hysteroscopy

## Abstract

**Background:**

The current research to the best of my knowledge is the first to compare the pregnancy outcome between ultrasound-guided tubal recanalization (UGTR) using a special fallopian tubal catheter, and office-based micrhysteroscopic ostial dilatation (MHOD) using the same tubal catherter in infertile women with previously diagnosed bilateral proximal tubal obstruction (PTO).

**Materials and Methods:**

This prospective study reported the pregnancy outcomes for 200 women in private infertility care center in Arafa hospital in Fayoum and in El Minya University Hospital in the period between January 2010 and October 2013 treated as outpatients for their bilateral PTO after the routine hysterosalpingography (HSG). A Cook’s catheter, special fallopian tubal catheter, were used to recanalize the blocked tubes in 100 women (group A) under UGTR, and the same Cook’s tubal catheter was used through 2mm microhysteroscope to cannulate both ostia using MHOD in another 100 women (group B). Pregnancy outcome was determined after the procedures for a 12-month period follow-up.

**Results:**

The number of the recanalization of PTO was not significantly different between two groups. As of the 200 blocked fallopian tubes in group A, 140 tubes (70%) were successfully recanalized by passing the ultrasound-guided special cannula, while 150 tubes (75%) were successfully recanalized in group B, using the same tubal catheter through a 2mm microhysteroscope. The cumulative pregnancy rate after the two procedures was not statistically different between two groups. It was 25.9% in group A, while it was 26.3% in group B, after a 12-month period follow-up.

**Conclusion:**

UGTR is highly recommended as the first step to manage infertile women due to PTO, as it is easier procedure; however, there is possible to obtain nearly similar results after MHOD.

## Introduction

Approximately 15% of couples are unable to conceive within a year of unprotected intercourse. A tubal condition known as proximal tubal obstruction (PTO) has been reported in 30% of infertile women. Fluoroscopically guided hysteroscopic tubal cannulation technique has been developed to improve diagnosis and treatment of tubal disease. For PTO, simpler cannulation technique has been also developed, guided by ultrasound or hysteroscopy (1,3). 

Recent advances in fiber optics have resulted in the development of transcervical tubal catheterization procedures with improved diagnostic and treatment accuracy of tubal disease with reduced risks, costs and morbidity. Fallopian tube recanalization can be performed with catheters or flexible guidewires under endoscopic, sonographic, or tactile guidance. Falloposcopy provides a possibility to visualize and grade endotubal disease, to characterize and document endotubal lesions, as well as to identify the segmental location of tubal pathology without any complications (4,8). 

Advances in hysteroscopy, including the introduction of small-caliber endoscopes, the flexible steerable hysteroscope and the use of video systems in monitoring hysteroscopic evaluations, have supported the application of office-based hysteroscopy for tubal cannulation both for diagnosing and treating cornual obstruction. Initial attempts with hysteroscopic proximal tube catheterization and balloon dilatation for recanalization have been proved intraoperatively successful in more than 80% of the cases (9,11). 

Sonographically-guided transcervical tubal catheterization and transcervical balloon tuboplasty may be successfully performed to diagnose and treat patients with PTO. It may be aided with color Doppler ultrasound-guided cannulation and transcervical wire tuboplasty, hysteroscopic/laparoscopic insertion of small intraluminal ultrasound transducers inserted into catheters with diameters of 3.5 F and 5 F during transcervical fallopian tube catheterization, and transvaginal sonographyguided trans-uterine cannulation of the tubes with the Jansen-Anderson embryo catheter (Cook, Australia) and injection of sterile fluid (2,4,8,12). 

The aim of the current study is to compare the pregnancy outcome in infertile women who were previously diagnosed with PTO using routine hysterosalpingography (HSG) between ultrasound-guided tubal recanalization (UGTR) and office-based micrhysteroscopic ostial dilatation (MHOD) procedures. 

## Materials and Methods

For this prospective study, 200 infertile women with a history of primary or secondary infertility were evaluated in private infertility care center in Arafa hospital in Fayoum and in El Minya University Hospital in the period between January 2010 and October 2013. The study protocol was approved by the ethics committee and institutional review board. The inclusion criteria were delayed conception for more than a year and bilateral PTO confirmed with routine HSG testing. Patients with unilateral PTO and other causes of infertility rather than PTO were excluded from the study. Recruited women were divided into two following groups: group A (n=100) using UGTR and group B (n=100) using the same tubal catheter through MHOD. 

## Ultrasound guided tubal recanalization procedure

**Fig.1:**
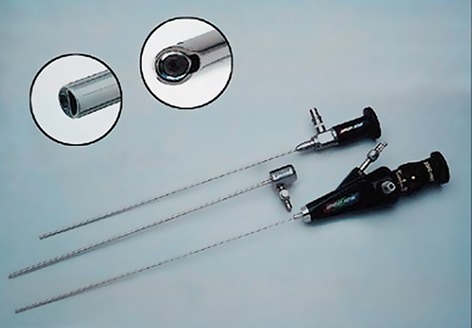
Two soft malleable fiberoptic microhysteroscopes with diameter of 2-mm used. The upper part is a telescope with operative channel of 0-degree and diameter of 2-mm. The lower part is a telescope with operative channel of 30-degree and diameter of 2-mm. The middle part includes the diagnostic sheath of the microhysteroscopic telescope.

Recanalization was performed during the early follicular phase of the menstrual cycle. The women were instructed to take one gram of azithromycin orally (Amoun, Egypt) before the procedure as an antibiotic prophylaxis, and 75 mg of diclofenac (Pharco, Egypt) was given intramuscularly on the day of recanalization, as an intra-operative analgesia. The women undergoing UGTR were placed in the lithotomy position with a partially full bladder in order to straighten the uterus. The cervix was exposed and the entire vagina was cleaned with betadine and draped. A Cook catheter (Cook South East Asia Pte Ltd., Singapore) was applied as in figure 1. 

The set included a speculum and a retinaculum, a flexible catheter, a guiding cannula, a tubal catheter, and a guidewire. After putting the speculum in place, the uterus was fixed by the retinaculum. Under transvaginal ultrasound scanning, the flexible guiding cannula was pushed forward into the uterus without dilation of the cervical canal and gently advanced to reach the cornua. The guiding cannula was then fixed to the retinaculum at a 45˚C and the tubal catheter was inserted in the cannula and advanced into the ostium until the obstruction was felt. 

**Fig.2:**
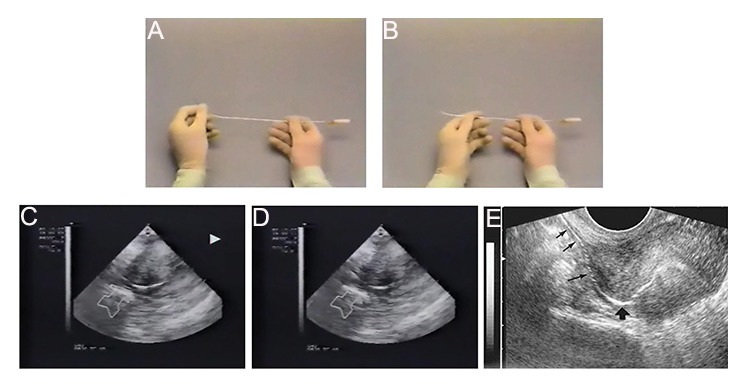
The cannula used and the ultrasound images of the ultrasound-guided tubal recanalization of proximal tubal obstruction (PTO): A. Cook’s catheter loaded with the guide wire, B. Cook’s catheter after exposure of the loaded guide wire, C. Ultrasound image of successful passage of the catheter within the right tube, D. Ultrasound image of successful passage of the catheter within the left tube and E. Ultrasound image of hydrotubation using saline and air after successfully recanalizing the proximaly blocked tube.

If there was resistance to the catheter advancement, the guidewire was threaded through the tubal catheter and the obstruction of proximal fallopian tube was removed. Tubal patency was then assessed by hydrotubation. Confirmation of tubal patency was achieved with observation of fluid accumulation in the cul-de-sac during the ultrasound scanning (Fig .2). 

The microlaparoscopic system consists of a light source, a high speed pneumoperitoneum device and full HD video camera (Sony, Japan). Furthermore a malleable fiberoptic scope, grasping forceps, scissors, and irrigator-aspirator (Olympus, Japan) with 2 mm in diameter were used. The instruments could be used by specially designed trocar (access needle, Ethicon, OH, USA). 

Premedication consisting of 0.5 mg of atropine sulfate and 1 mg/kg of midazolam (Cairo medical industry, Egypt) was given intramuscularly. One mg/kg of fentanyl (Cairo medical industry, Egypt) followed with 1.5 mg/kg of ketamine was intravenously administered through a drip infusion (Fig .1). The patient was place in a lithotomy position. An access needle was inserted through a small incision created in the subumblical region using the closed method, after being locally infiltrated with xylocaine (Sigma, Egypt), a local anesthesia. 

Pneumoperitoneum was introduced with carbon dioxide gas. Other access instruments were then inserted into both sides of the hypogastric regions under microlaproscopic guidance. Four ml/port of 0.25% bupivacaine (Sigma, Egypt) were locally injected at the trocar insertion sites in advance. Subsequently, a micrograsper was used to expose a fimbrial tube at the time of hydrotubation. Four ml of 0.5% lidocaine was sprayed over the diaphragmatic vault. After the removal of the trocars, 5 mL of bupivacaine was then injected into the subcutaneous (SC) tissue of the insertion sites. 

## Office microhysteroscopic ostial dilatation

**Fig.3:**
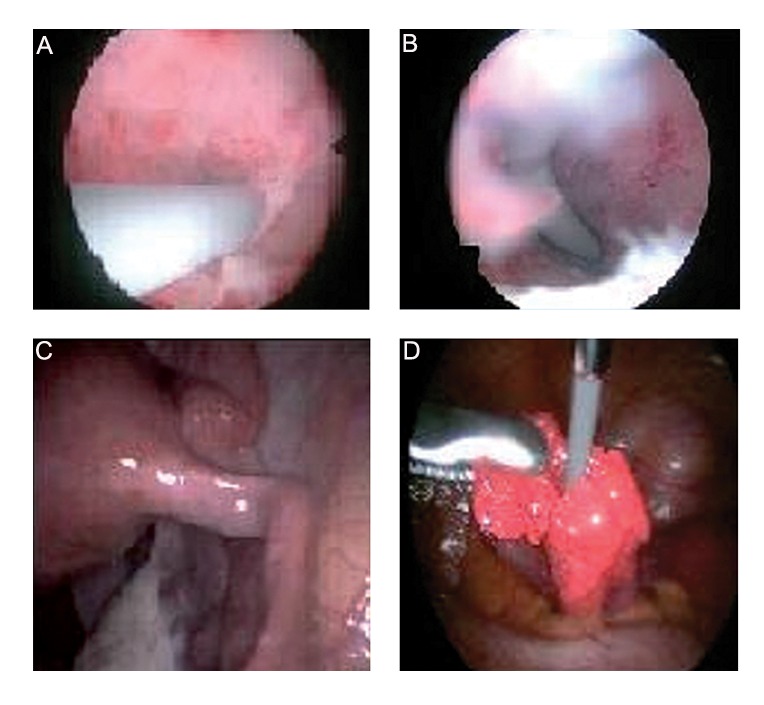
These images are produced after the combination of micrhysteroscopic and microlaparoscopic ostial recanalization for proximal tubal obstruction (PTO): A. Tip of the ureteric catheter directs to the visualized ostial opening, B. Catheter successfully cannulates within the tubal lumen, C. Microlaparoscopic image shows the picture of the cannulated tube and D. Microlaparoscopic image shows the appearance of the tip of the Cook’s catheter beyond the fimbrial ostium after successful recanalization.

All office-based microhysteroscopies were performed using a malleable fiberoptic microhysteroscope with operative channel of 30-degrees and diameter of 2-mm for the use of 5-6 F Cook’s catheter (Cook, Australia) (Fig .1). The catheter was placed through the built in operative channel and proceeded in order to be visualized at the tip of the hysteroscope. After visualization of both ostia, the catheter was pushed slowly with moderate degree of pressure relative to the resistance faced. Confirmation of successful recanalization would be made with the concomitant use of 2-mm microlaparoscope. Then methylene blue (Naser Biomedical Company, Egypt) was used for chromopertubation as a definite proof of tubal recanalization. Typically, less than 1 liter of normal saline was used as the distention media for procedures (Fig .3). 

## Follow-up of pregnancy outcome after both procedures

Women with successful recanalized tubes were followed up for the cumulative pregnancy rate (CPR) for a 12-month period for each group of women studied. Pregnancy outcome was correlated to the unilaterality or bilaterality of the tubes recanalized. Pregnancy complications were also evaluated, especially the incidence of developing ectopic pregnancy. Women with unsuccessful recanalized tubes who never sought another treatment for their blocked tubes were also followed up for the same period. 

## Results

Of the 200 women diagnosed by HSG as having bilateral PTO, 40 women were younger than 20 years, 141 were aged between 21 and 30 years, and 19 were aged between 31 and 40 years, while 76 (76%) had primary infertility and 48 (24%) had secondary infertility. 

In between recruited women for UGTR in group A, 140 (70%) of the 200 tubes were recanalized successfully. Successful recanalization was bilateral in 20 women [n=40 (20%)] and unilateral in 65 women [n=65 (65%)]. Recanalization failed in the remaining 15 women [n=30(15%)] (Table 1). While use of MHOD in group B succeeded to canalize 150 (75%) tubes, which was bilateral in 20 women [n=40 (20%)] and unilateral in 70 women [n=140 (70%)] (Table 1). 

In group A, the women with one or two successful recanalized tubes were followed up for CPR for 12 months, where 17 (20%) conceived within 6 months and 22 (25%) after another 6 months. While, in group B, 20 conceived (22.2%) after 6 months and 25 (27.7%) after another 6 months. Therefore, CPR was higher after two tubes recanalized (25%) in group A versus (32%) in group B, while CPR was 25% in group A versus 29% in group B after only one tube recanalized (Table 2). Ten and five of these conceptions were spontaneous in group B and group A, respectively, while the other conceptions occurred after ovulation induction and monitoring. Two and three ectopic pregnancies occurred after complicated UGTR and MHOD, respectively. Follow-up of cases failing to be recanalized showed another case of ectopic pregnancy. No serious complications were encountered. 

Group B gained more benefits from combined of- fice-based microlaparoscopy and microhysteroscopy procedures. Congenitally absent, rudimentary or highly suspended tubes were diagnosed in 5 cases. Peritubal and peri-ovarian adhesions were observed in 6 cases and managed in the same session. Mild to moderate degree of pelvic endometriosis were diagnosed in 3 cases, and managed in the same session. Both tubes failed to be recanalized in the previously diagnosed pelvic abnormalities. Microhysteroscopy were diagnosed in 3 cases with endometrial polyps, of which two and three cases had polypoid endometrium and intrauterine adhesion, respectively. All those abnormalities were managed in the same session. 

**Table 1:** Numbers of successful and unsuccessful recanalized tubes after the two studies procedures


	Procedure	
	UGTR (n=100)	MHOC (n=100)

Tubes successfully recanalized	140 (70%)	150 (75%)
Bilaterally	40 (23.5%)	50 (26.3%)
Unilaterally	130 (65%)	140 (70%)
Tubes unsuccessfully recanalized	30 (15%)	10 (5%)


UGTR; Ultrasound guided tubal recanalization and MHOC; Microhysteroscopic ostial cannulation.

**Table 2:** Comparison of pregnancy outcome between both studied procedures for tubal recanalization


Pregnancy Outcome	UGTR	MHOD

CPR after 6 months	20% (n=17)	21% (n=20)
CPR after 12 months	25.9% (n=22)	26.3% (n=25)
CPR after bilateral TR	25% (n=5)	32% (n=8)
CPR after unilateral TR	26.1% (n=17)	29.3% (n=23)
Spontaneous pregnancy	22% (n=5)	40% (n=10)
Ectopic pregnancy	13% (n=3)	8% (n=2)


CPR; Cumulative pregnancy rate, UGTR; Ultrasound guided tubal recanalization, MHOD; Micrhysteroscopic ostial dlilatation and TR: Tubal recanlaization.

### Statistical analysis

The accuracy of HSG for PTO was assessed by the chromopertubation under microlaparoscopy. The success rate of recanalization, the pregnancy rate (PR) following both procedures, and the time taken to achieve pregnancy were investigated. Student’s t test and the Mann-Whitney U test were used. A value of P<0.05 was considered statistically significant. 

## Discussion

Tubal disease is the cause of subfertility in approximately 30% of women and 25% of these are due to PTO. PTO has been a diagnostic and therapeutic dilemma since its recognition more than 100 years ago. It can occur in either the intra-mural segment or the uterotubal junction, and is the result of tubal spasm or transient occlusion by mucus plugs in up to 40% of women. Proximal, distal and peritubal damage can be caused by a number of pathologic processes, such as inflammation, endometriosis and surgical trauma (13,16). 

Obliterative fibrosis has been observed as the most common histologic tubal abnormality by both Wiedemann et al. (16) and Fortier and Haney (17), followed by salpingitis isthmica nodosa. 

The highest incidence of salpingitis isthmica nodosa (SIN) has been reported 60% by Papaioannou et al. 

(5,6), while chronic tubal inflammation was 70.59% by Zhang et al. (15). On the basis of their observations regarding the pathologic spectrum of uterotubal junction (UTJ), Fortier and Haney (17) demonstrated that there are multiple distinct histologic patterns and intra-abdominal findings that do not predict the histology of the UTJ pathology (18,20). 

Minimally invasive transcervical tubal catheterization procedures provide an excellent alternative to invasive and expensive surgical procedures and assisted reproductive technology (ART) for the diagnosis of tubal disease and treatment of minimally diseased proximal fallopian tubes. Fallopian tube recanalization (FTR) can be performed with catheters, flexible atraumatic guidewires or balloon systems under endoscopic (falloposcopy/hysteroscopy/laparoscopy), sonographic, fluoroscopic or even with tactile guidance (21,22). 

The current work aimed to evaluate the efficacy of a simple, easy, and reliable tool for recanalization of PTO in group A and to compare it to a more complicated procedure in group B. Therefore, the main points worthy of attention are that there are insignificant differences in success level for recanalizing the blocked tubes and developing PR after the two procedures. Moreover the minimal degree of intervention and the absence of different types of anesthesia were observed in this procedure. 

Initial attempts with hysteroscopic proximal tube catheterization and balloon dilatation for recanalization proved intraoperatively successful in more than 80% of the cases. Under laparoscopic guidance, the hysteroscopic approach enables tubal cannulation and evaluation of the entire pelvis. Treatment of additional problems affecting the fallopian tubes, particularly adhesions and endometriosis is possible. Moreover laparoscopy helps to monitor the procedure and offers to assess tubal patency, leading to the ability to observe the utero-tubal junctions (UTJs) directly by hysteroscopy and to provide an excellent approach for tubal cannulation, but there is still a need for anesthesia (11,15,23,25). 

In the current work, extra findings diagnosed (e.g. endometrial polypi, adhesions, and infection) during the concomitant office-based micrhysteroscopy and microlapaproscopy procedures in group B never added much to the final prognosis of the case of PTO, as the tubes in those women failed to be recanalized during the combined procedures. So, those cases with extra findings never affected the final pregnancy outcome. We recommend shortlisting the cases with PTO who failed to be recanalized after UGTR to be recruited for the combined office-based micrhysteroscopy and microlaparoscopy procedures, which can be considered as the second recommended step of management protocol. 

Li et al. (9) described a soft and rigid operating fiberoptic hysteroscope [operative channel diameter (OD) 4.8 mm] that can be used clinically for transcervical tubo-cornual recanalization for the management of cornual occlusion. The functional part of the telescope consists of three sections: a soft, flexible front section; a rigid rotating middle section; and a semi-rigid, self-retaining rear section offering advantages of an easy, close and direct approach to the intrauterine target, usually with no cervical dilation or anesthesia, with the operator in a comfortable position and without reported complications. This new hysteroscope has proved to be a very useful tool for the treatment of intrauterine lesions in the theater or an office setting. Clinical results in 1503 women who underwent this panoramic, televised fiberoptic hysteroscopy without cervical dilation suggest that the soft and rigid structure of the diagnostic fiberoptic hysteroscope offers advantages over rigid scopes or conventional fiberscopes with full-length soft, malleable parts (15,18,20,26). 

Additional findings diagnosed during the microhysteroscopic procedure in group B could be also checked and diagnosed in group A, meaning that the use of an electrolyte solution, like regular saline for uterine inflation during the tubal recanalization procedure, which is added for confirmation of successful tubal recanalization in order to visualize the hydrotubated recanalized tube/tubes in addition to test of the accumulated fluid in the Douglas pouch during the same ultrasound session. So it is of importance to consider office-based combined microhysteroscopy and microlaparoscopy procedures in order to treat those abnormalities mentioned before, as a second line of management. 

Combined hysteroscopic tubal cannulation with selective salpingography under fluoroscopic guidance has been previously reported as a safe and simple diagnostic method that has also been used to identify and to treat successfully the interstitial fallopian tube obstruction. However, compared with the other hysteroscopic cannulation techniques, the addition of selective salpingography under fluoroscopic guidance to hysteroscopic tubal cannulation appears to yield the lowest patency and PRs. A systematic review of observational studies showed that hysteroscopic tubal cannulation was associated with a higher PR (49%) than salpingography and tubal cauterization (21%) in women with PTO (20,22,27). 

The relatively higher success rate of recanalizing tubes after the second procedure in group B could be justified to the more accurate visualization of both ostia before passing the Cook’s catheter, which definitely facilitated the successful cannulation, in contrast to the ultrasound guided cannula which pushed to the direction of the ostia, without definitely visualizing both ostia. Moreover increased intrauterine pressure, developed from the uterine distension media used during the MHOD procedure, added more to the higher success in recanalizing the proximally blocked tubes in group B. In group A, the previously mentioned optional hydrotubation could also increase the success of recanalizing the PTO during UGTR. 

The relatively higher PR developed after the second procedure in group B could be due to more than a factor. Firstly the successful recanalization of the blocked tubes without creating a new false tract was observed more in the second procedure, which allowed for proper visualization of both ostia in contrast to the first procedure. Secondly microhysteroscopic uterine irrigation with the distension fluid media studied before was showed to lead to an increase in the PR after hysteroscopy. Thirdly the ability to mange intrauterine abnormalities during the microhysteroscopic session could also lead to this relatively higher PR. 

Combined laparo-hysteroscopic tubal cannulation with or without guidewire cannulation in previous studies has yielded an average recanalization success rate of 76%, with an average intrauterine PR as high as 39%. Using combined laparoscopy and hysteroscopic tubal cannulation, Das et al. (1) concluded that hysteroscopic cannulation of the fallopian tube is a safe diagnostic procedure that can be used to identify those patients with true proximal occlusion, and may also serve as a therapeutic procedure in some of these patients. However, conception in their study was achieved after tubal cannulation and adjunctive distal tubal surgery, confounding the results. Zhang et al. (15). performed combined laparo-hysteroscopic cannulation of the proximal oviduct with a flexible guidewire to evaluate and treat intramural fallopian tube obstruction, and also concluded that this procedure is an effective method to manage PTO (10,15,22,28). 

In this study, another tool for tube recanalization was applied, like falloposcopy, which provides a unique possibility to accurately visualize, characterize and grade endotubal disease; identifies the segmental location of tubal pathology without complications; objectively classifies the cause of proximal tubal obstruction; as well as guides future patient management in contrast to laparoscopy and HSG that are often associated with poor or misdiagnosis of proximal tubal obstruction; however, these procedures require higher advanced instrumentation and more expert personnel. Non-hysteroscopic transuterine falloposcopy, using the linear eversion catheter, is a well-tolerated technique that can be performed in an outpatient clinic with high rates of luminal cannulation and visualization and a good predictive value for future fertility (8,12,16,20,26). 

Guidewire cannulation in group A yielded much lower PRs as compared with the study of Zhang et al. (15), in which they used different catheter techniques, whereas their findings showed similar tubal patency rates. Considering the associated risk factors into account, tubal recanalization procedures are contraindicated in presence of florid infections, genital tuberculosis, obliterative fibrosis, long tubal obliterations that are difficult to bypass with the catheter, severe tubal damage, male subfertility and previously performed tubal surgery. Distal tubal obstruction is not amenable to catheter recanalization techniques, while tuberculosis, salpingitis isthmica nodosa, isthmic occlusion with club-changed terminal, ampullar or fimbrial occlusion and tubal fibrosis have been cited as reasons for recanalization failure (29). 

Ultrasound-guided cannulation is essentially a development of the selective fluoroscopic fallopian tube cannulation method. Ultrasound replaces fluoroscopy as a mean to observe tubal flushing and makes repeated cannulation possible. Although the ultrasound-guided method requires expert handling of the catheter and guidewire during cannulation, it has several advantages over its fluoroscopic precursor. It is less expensive, more readily available, applicable for outpatient basis, noninvasive, diagnostic, therapeutic, as well as less painful. Coaxial catheter systems have long used, with consistent success, for the transcervical cannulation of fallopian tubes under hysteroscopy, fluoroscopy, ultrasonography, or tactile sensation (17,26,28). 

## Conclusion

UGTR is a simple, easy, feasible, and effective procedure to recananlize PTO with significantly low costs as compared to MHOD, which although had higher success in recanalizing PTO that was followed with higher pregnancy outcome, in addition to the extra findings diagnosed and managed in the same session; however, this difference is still not significant, while an operative intervention, like anesthesia, is necessary. Those facts could support the recommendation to consider UGTR as the first step in the management protocol of PTO before any further advanced surgical or ART. Still future studies are required to assess the risk factors associated with PTO, the relative success of tubal recanalization, and subsequent pregnancy outcome after the procedure. 
